# Primary tumor resection in colorectal cancer patients with unresectable distant metastases: a minireview

**DOI:** 10.3389/fonc.2023.1138407

**Published:** 2023-04-27

**Authors:** Junge Bai, Ming Yang, Zheng Liu, Sergey Efetov, Cuneyt Kayaalp, Audrius Dulskas, Darcy Shaw, Xishan Wang

**Affiliations:** ^1^ Department of Colorectal Surgery, National Cancer Center/National Clinical Research Center for Cancer/Cancer Hospital, Chinese Academy of Medical Sciences and Peking Union Medical College, Beijing, China; ^2^ Clinic of Coloproctology and Minimally Invasive Surgery, I.M. Sechenov First Moscow State Medical University (Sechenov University), Moscow, Russia; ^3^ Department of Gastrointestinal Surgery, Yeditepe University, Istanbul, Türkiye; ^4^ Department of Abdominal and General Surgery and Oncology, National Cancer Institute, Vilnius, Lithuania; ^5^ Faculty of Medicine, Institute of Clinical Medicine, Vilnius University, Vilnius, Lithuania; ^6^ Colorectal Surgery Associates, Kansas City University, Kansas, MO, United States

**Keywords:** colorectal cancer, primary tumor resection, neoplasm metastasis, survival, chemotherapy

## Abstract

Colorectal cancer (CRC) is the second most common cause of cancer-related death among both men and women worldwide and the third most common cancer overall. About 20% of patients diagnosed with CRC were discovered to have distant metastatic lesions, the majority of which were located in the liver. For the optimum treatment of CRC patients with hepatic metastases, interventional radiologists, medical oncologists, and surgeons must all collaborate. The surgical excision of the primary tumor is an important part of CRC treatment since it has been found to be curative in cases of CRC with minimal metastases. However, given the evidence to date was gathered from retrospective data, there is still controversy over the effectiveness of primary tumor resection (PTR) in improving the median overall survival (OS) and quality of life. Patients who have hepatic metastases make up a very tiny fraction of those who are candidates for resection. With a focus on the PTR, this minireview attempted to review the current advancements in the treatment options for hepatic colorectal metastatic illness. This evaluation also included information on PTR’s risks when performed on individuals with stage IV CRC.

## 1 Introduction

Colorectal cancer (CRC) is the third most common kind of malignancy to be diagnosed. It is estimated that by 2020, there will be more than 1.9 million new cases and 935,000 deaths of CRC, making it the second leading cause of death from cancer overall, in both men and women (1). Incidence and mortality rates for CRC are reported to be highest in North America, Europe and Oceania (2), and the numbers have increased due to changes in diet and lifestyle, notably in China (3). More than half of CRC patients develop metastases, of whom 20% had distant metastatic lesions at the initial diagnosis or during the treatment, predominantly in the liver (4, 5). For the localized stage, the 5-year relative survival rate can reach to 90%, but it is less than 10% for the remote stage (1). Currently, long-term treatment goals for CRC patients with distant metastases focus on improving overall survival (OS) and quality of life.

Surgeons, medical oncologists, and interventional radiologists must all work together to develop a multidisciplinary strategy for the best therapy for CRC patients with hepatic metastases. For CRC patients with few metastases, surgical removal of the main tumor is essential and has been shown to be curative. Only 10–20% of stage IV CRC patients, however, have surgical indications for treatment since the majority (75–90%) have untreatable distant metastases (6, 7). As a result, the National Comprehensive Cancer Network (NCCN) guidelines do not typically advise patients with stage IV CRC to go through primary tumor resection (PTR) as a form of curative surgery (8). PTR is only taken into consideration for patients with stage IV CRC that is resectable if all metastatic lesions can be removed concurrently (9).

For managing the main tumor-related symptoms and side effects (such as blockage, perforation, or refractory bleeding), surgeons have traditionally advised PTR (10). However, currently, several systemic chemotherapies and biological targeted agents (e.g., 5-fluorouracil, oxaliplatin, irinotecan, cetuximab) are available and have become the first-line regimens for metastatic colorectal cancer during the past ten years. For example, fluorouracil-based induction chemotherapy has a significant effect on primary tumor and liver metastasis (11). The monoclonal antibody bevacizumab in conjunction with oxaliplatin-based chemotherapy has been found to significantly increase the metastatic CRC patients overall survival from 6 months to 24 months in a randomized phase III trial (12). As reported by two population-based investigations using the Surveillance, Epidemiology, and End-Results (SEER) database, PTR was gratuitously performed in almost 70% of CRC patients despite the increased effectiveness and widespread usage of these medications (6, 13). Early studies concluded that PTR improved median OS and quality of life in patients with stage IV CRC. However, these studies were retrospective, single-center, observational, lacked corroboration from prospective randomized controlled trials, with selection bias and unknown confounders, and may have weakened the robustness of the conclusions. In addition, the risks associated with PTR performance (e.g., post-PTR complications and mortality) have not been adequately assessed. Therefore, the implementation of the non-curative PTR as an initial treatment option requires a systematic assessment in terms of its benefits and risks. If the surgery of primary tumors is ineffective in prolonging survival or reducing serious postoperative complications, patients are going to be exposed to a higher risk of morbidity, mortality and uncalled-for expenses. With a particular focus on the efficacy and safety of PTR, this minireview attempted to develop an extensive specialization in the treatment of patients with viscus metastases from CRC. Additionally, this review aimed to spot the determinants which may influence the choice creating of aid suppliers to perform PTR. This will help to inform the future practice and figure out in whom PTR is of benefits.

## 2 The advantages of initial tumor removal in stage IV CRC patients

### 2.1 Effectiveness and efficacy of PTR in stage IV CRC patients

The effectiveness of PTR in CRC patients with unresectable metastases remains polemic (14), with some studies advised that PTR will considerably improve the survival and quality of life, while others have failed to find a significant difference in its survival benefit. For instance, a Japanese randomized controlled trial (RCT) evaluated the role of PTR prior to the initiation of chemotherapeutical agents in improving OS in CRC patients. However, no statistically significant OS was found between the patients (median OS 25.9 months for the PTR group vs. 26.4 months for the non-PTR group, P>0.05) (15) (**Tables 1**, **2**). The latest CAIRO4 Phase 3 Randomized Clinical Trial (16) investigated 60-day mortality in patients with CRC and found that patients randomized to PTR followed by fluoropyrimidine-based chemotherapy with bevacizumab had higher mortality compared to the control group (systemic treatment, consisting of fluoropyrimidine-based chemotherapy with bevacizumab). Both of these studies have the advantage of being RCTs, and both concluded that PTR may not be beneficial in improving survival in CRC patients, but both studies have the commonality of small sample size, with a total sample size of only 361.

**Table 1:** Characteristics of selected studies assessing the impact of PTR on survival or survival benefit in unresectable stage IV CRC.

Author and years	Country	Study design	Cases (n)	PTR/Non-PTR (n)	Median age (years)		Male proportion (%)	Median follow-up (months)
					PTR group	Non-PTR group		
Kanemitsu et al. (15) 2012-2019	Japan	RCT	165	81^a^/84 ^b^	65 (59-69) ^a^	65 (50-71) ^b^	90 (54.5)	22.0
van der Kruijssen et al. (16) 2012-2019	Denmark、Netherland	RCT	196	97/99	64 (59-70)	65 (57-70)	112 (57)	2
Lam-Boer et al. (17) 2008-2011	Netherland	Retrospective study	10,371	2,746/3,345	NA, (60-75)	NA, (60-75)	3493 (57.3)	17.6 ^c^
Ahmed et al. (18) 2006-2010	Canada	Retrospective study	569	313/256	68 (33-95)	70 (30-95)	335 (59)	11 (2-26)
Doah et al. (19) 2001-2018	Korea	Retrospective study	146	98/48	69 (58-77)	66.5 (62-75)	78 (53.4)	Not reported
Kawamura et al. (20) 2008-2015	Japan	Retrospective study	616	414/202	69 (60-77)	67 (60-74)	383 (62.2)	18 (8.4-29.7)
Niitsu et al. (21) 2007-2013	Japan	Retrospective study	57	42/15	61.5 (54-70.5)	63 (48-65)	15 (26.3)	Not reported
Urvay et al. (22) 2009-2016	Turkey	Retrospective study	215	139/76	59 (22-85)	62 (27-86)	136 (63.2)	24.6 (1.1-105.5)
van Rooijen et al. (23) Before 2017	Multicenter	Retrospective study	3,423	2,713/710	Not reported	Not reported	2146 (62.7)	Not reported
Xu et al. (24) 2006-2012	America	Retrospective study	31,310	6,888/11,741	73 (62-82)	63 (55-73) ^b^	1835 (49.1)	Not reported
Zhang et al. (25) 2007-2013	China	Retrospective study	194	125/69	NA, (50-75)	NA, (50-75)	1319 (67.4)	Not reported

RCT, randomized controlled trial; PTR, Primary Tumor Resection; NA, not available; a, PTR + chemotherapy; b, chemotherapy alone; c, for the surgical group, the median follow-up was 17.6 months, but for the systemic treatment group, it was 13.9 months.

**Table 2:** The survival advantage associated with PTR in non-resectable stage IV CRC.

Author	Survival (months, range)/Mortality (%)		95% CI or P value
	PTR group	Non-PTR group	
Kanemitsu et al. (15)	25.9 (19.9-31.7)	26.4 (21.9-32.1)	HR 1.11; 95% CI: 0.78-1.58
van der Kruijssen et al. (16)	2% (1%-7%)	10% (5%-18%)	P=0.048
Lam-Boer et al. (17)	16.2 (15.4-17.1)	12.1 (11.7-12.5)	HR 0.44; 95% CI: 0.35-0.55 P<0.001*
Ahmed et al. (18)	18 (15.4-20.6)	4 (2.6-5.4)	HR 0.44; 95% CI: 0.35-0.56 P<0.001*
Doah et al. (19)	18 (range NA)	15 (range NA)	HR 0.61; 95% CI: 0.40-0.94 P=0.152
Kawamura et al. (20)	23.9 (12.2-39.9)	12.3 (6.2-23.8)	HR 0.51; 95% CI: 0.42-0.64 P<0.001*
Niitsu et al. (21)	23.9 (range NA)	13.4 (range NA)	HR: 0.57^#^ P=0.093
Urvay et al. (22)	29.6 (range NA)	14.3 (range NA)	HR NA P<0.001*
van Rooijen et al. (23)	22.2 (range NA)	16.4 (range NA)	HR: 0.63^#^
Xu et al. (24)	6 (range NA)	13 (range NA)	HR NA P<0.0001*
Zhang et al. (25)	22 (range NA)	14 (range NA)	HR: 0.57^#^ P=0.009*

RCT, randomized controlled trial; PTR, Primary Tumor Resection; CI, confidence interval; NA, not available; * significantly difference; ^#^Converted based on the HR of the control group.

In addition to the two RCTs mentioned above, we included nine other retrospective observational studies (17–25). Almost all of these studies support the survival benefit of PTR to patients (**Table 2**). Of a total of approximately 30,000 patients in these nine studies, only the study by Xu et al. found no significant survival benefit in the PTR group (24). Lam-Boer et al. (17) and Doah et al. (19) performed a propensity score matching analysis (so-called *post hoc* randomization) of the included population to minimize selection bias, and both studies confirmed the advantage of PTR in improving survival in patients with in unresectable stage IV CRC. Another retrospective longitudinal study conducted by Kim et al. found that 103 patients with nonlocally advanced tumors who underwent PTR had an improved overall survival by 17.8 months (95% CI 16-19.5 months, P<0.05) (26). The overall survival of patients who got PTR improved by 7.76 months (95% CI 5.96-9.56 months, P 0.05) according to a meta-analysis of 148,151 patients from 56 retrospective studies (27). However, a high heterogeneity was noted in this study. Overall, PTR has the potential to improve patient survival in larger samples of clinical studies, but requires validation by RCTs.

PTR alone can improve survival irrespective of other treatments. In the study of Urvay et al., 215 patients who had stage IV unresectable metastatic CRC had a median overall survival of 29.56 months, whereas the patients who did not get PTR had a median OS of 14.25 months (P=0.001). OS was 35.6 months in the PTR group whereas 22.2 months in the non-PTR group who only received chemotherapy (P=0.002). For the surgical group, the median progression-free survival (PFS) was 9.85 months, but for the non-surgical group, it was 7.06 months (P=0.001) (22). These trials provided more evidence that PTR can increase survival for metastatic, unresectable CRC. In an RCT carried out across multiple centers, in a sample of 48 patients with advanced stage IV CRC, the PTR group (n=26) had a significantly higher 2-year cancer-specific survival rate than the upfront chemotherapy group (n=22) (72.3% vs. 47.1%, P=0.049) (28). The two-year PFS rate was not significantly different between the two groups (69.5% vs 44.8%, P=0.058), which may have been a result of the small sample size. Similar conclusions were reached by a systematic review and meta-analysis encompassing 159,991 people (PTR n=94,745; primary tumor intact [PTI] n=65,246): the PTR group had a significantly longer OS (7.46 months, HR 0.58, P<0.0001) than the PTI group, but there was also a significant between-group heterogeneity (P<0.0001). A prolonged PFS (HR 0.76, P<0.0001; MD 1.67 months, P<0.0001) and cancer-specific survival (HR 0.44, P<0.0001; MD 10.01 months, P<0.0001) were also associated with PTR (29). The median OS for 616 patients with advanced CRC (PTR n=414; non-PTR n=202) was 23.9 months in the PTR group whereas 12.3 months in the non-PTR group, according to a further retrospective multicenter analysis (P<0.001). The interquartile range for the PTR group was 12.2-39.9 months (adjusted HR 0.51; 95% CI 0.42–0.64; P<0.001). PTR was substantially linked to a better OS. This strongly suggests that PTR contributes to an improved prognosis irrespective of the chemotherapy regimens.

PTR combined with postsurgical systemic chemotherapy may be more beneficial for patients with advanced stage IV CRC who are asymptomatic than either treatment alone (i.e., PTR alone or chemotherapy alone). The cumulative 5-year overall survival rate was 28.3% for patients who received PTR plus chemotherapy, 17.6% for patients who only received chemotherapy, 15.9% for patients who only underwent PTR, and 9.1% for patients who were not treated, according to an analysis of retrospective cohort studies using the American National Cancer Data Base (n=31,310). The median OS (23 months) for patients who got PTR and then chemotherapy was likewise much longer than it was for individuals who underwent chemotherapy alone (13 months), PTR alone (6 months), or received no treatment at all (2 months) (24).

### 2.2 PTR might decrease the emergency surgery during chemotherapy

Stage IV patients who had initial therapy and had an unresectable tumor experienced a 22% incidence of complications attributable to the primary tumor. In this situation, 87% of patients have to discontinue their chemotherapy regimens for approximately 4 weeks and seek emergency surgery (24), which in turn put the patients at a higher risk of having unexpected complications (30%) or hospital mortalities (8.5%) (30). Furthermore, research has shown that persons who experienced initial tumor difficulties during chemotherapy were more likely to have a colon cancer prognosis that was concerning (31). According to a secondary database analysis conducted in the western Netherland, the post-surgery mortality rate on the 30^th^ day was only 1.5%. However, it increased to 8.8% after the onset of symptoms (32). According to other research, if the main tumor is not removed during the initial therapy, 7–22% of patients will need emergency surgery or intervention (33–35). A non-randomized prospective controlled study by Wang et al. demonstrated that PTR reduced the incidence of serious clinical events and improved patients’ quality of life, whereas the group of patients not treated with PTR could require emergency surgery due to significant primary tumor-related complications (including bleeding, perforation, etc.) (36).

## 3 Factors contributing to a better prognosis for stage IV CRC patients undergoing PTR

**Table 1** shows the baseline characteristics of individuals who received PTR or non-PTR in a few trials. The median age of the patients ranged from 60 to 75 years, and no appreciable differences were discovered between the studied groups. The majority of the studies had a higher proportion of male participants, except for Niitsu et al. (21) who had more female than male patients, but there was no statistically significant difference in gender between individuals who underwent surgery and those who did not.

Identifying which CRC patients with metastases may benefit from PTR is of profound importance. Previous studies conducted multivariate analyses and the following were identified as independent factors affecting prognosis: age (older patients have poorer prognosis), American society of anesthesia (ASA) score/WHO-PS (the lower the score, the better the prognosis. E.g: ASA score < 3, WHO-PS < 2), preoperative CEA levels (high levels indicate a poor prognosis), primary tumor location, size, and differentiation, tumor burden, the extent of hepatic metastases (liver involvement < 50% with a better prognosis), peritoneal dissemination, and extra-hepatic metastases(the smaller the extent of metastasis and the extent of the primary tumor, the better the prognosis) (10). As for the primary location of the tumor, Zhang et al. found that patients with left-sided colon cancer may benefit from PTR (25). Other variables that impacted prognosis but were less frequently noted were serum albumin, alkaline phosphatase levels, lymph node involvement, ascites, the number of metastatic sites, and the use of targeted therapy (10). In summary, surgeons should consider these prognostic factors when making the decision to perform PTR. Of course, PTR is obviously not recommended for patients with contraindications, including patients with a high burden of advanced metastatic tumors, etc.

## 4 Risks of PTR in CRC patients with incurable metastases

The most common complications after PTR include anastomotic leakage, intestinal obstruction, wound infection, adaptive immune suppression secondary to anesthesia, and blood transfusion. Systematic inflammatory response and homeostasis disorder after PTR can further promote immunosuppression and accelerate the growth of metastases (27, 37). Postoperative problems are associated with longer hospital admissions, delayed recovery, and later initiation of systemic therapy, all of which have a poor impact on the physical state, prognosis, and survival of patients who received PTR.

The severe complications caused by PTR can negatively influence the prognosis of patients. Anastomotic leak, obstruction, and wound infection were the most frequent severe postoperative complications in a multicenter retrospective cohort study carried out in Japan, which included 93(9.6%) patients (n=966). These complications were defined as National Cancer Institute Common Terminology Criteria for Adverse Events v3.0 [CTCAE] grade 3 or 4. Major problems were characterized as CTCTAE grades 1 or 2, and patients who had them had a substantially worse prognosis than those who did not (HR1.62, 95% CI1.21-2.18, P<0.01) (38). As a result, some oncologists advocate using chemotherapy as the first-line treatment option, as they are concerned about the impact of postoperative complications, particularly when patients experience a deteriorating physical condition (e.g., weight loss and malnutrition) after surgery. However, long-term chemotherapy may lead to higher toxicity compared with patients who underwent PTR. Therefore, the best therapy for people with stage IV CRC must thus be chosen by weighing the risks and advantages.

## 5 Conclusions

The prognosis is poor for patients with metastatic colorectal cancer, and they are more likely to experience potentially fatal tumor-related complications such as blockage, perforation, and bleeding. The advantages of PTR, even though they were performed in around 70% of patients with metastatic CRC, were still not completely clear. Previous reviews on PTR in CRC patients have reported high rates of postoperative mortality and morbidity associated with PTR (10, 39). These studies ignore the heterogeneity of the included studies, which may result in misunderstanding and bias towards PTR. In this study, we focused on reviewing the survival benefit of PTR for stage IV CRC patients in several previous retrospective studies, and we emphasize that the potential benefits of PTR should be re-examined. We synthesized clinical studies from the last decade and came to similar conclusions as Pędziwiatr, Liang et al. that PTR may actually be beneficial, mainly in terms of prolonging survival (40, 41). We also highlight the heterogeneity (selection bias and unknown confounders may distort the conclusion) of current studies, present some of the key factors affecting patient prognosis, and advocate that surgeons pay attention to this evidence and carefully assess the risks and benefits of surgery before proceeding. Finally, PTR is still widely performed and high postoperative complications and mortality remain a fact, but there are still no large sample, multicenter RCTs to validate the role of PTR, especially in the current era of targeted therapies (biologic agents, bevacizumab or cetuximab), thus high quality RCTs are needed to compare its effectiveness with alternative treatment options.

## Author contributions

JB and MY completed the first draft, CK, AD, DS and XW collected data and drew tables, and ZL and SE revised the article. All authors contributed to the article and approved the submitted version.
